# Field-based physical fitness assessment in preschool children: A scoping review

**DOI:** 10.3389/fped.2022.939442

**Published:** 2022-08-02

**Authors:** Dandan Ke, Remili Maimaitijiang, Shaoshuai Shen, Hidetada Kishi, Yusuke Kurokawa, Koya Suzuki

**Affiliations:** ^1^School of Public Health, Fudan University, Shanghai, China; ^2^Graduate School of Health and Sports Science, Juntendo University, Chiba, Japan; ^3^School of Education and Welfare, Aichi Prefectural University, Aichi, Japan

**Keywords:** health-related fitness, motor skill-related fitness, physical performance, physical activity, growth and development, early childhood, physical fitness test battery

## Abstract

Physical fitness, which can be measured using various health- and skill-related components, is an important indicator of child development and health status. This study undertakes a scoping review on physical fitness assessment methods in preschool children to summarize the most widely used field-based physical fitness batteries and specific test items for preschool children. A search of the literature in English was undertaken using two major electronics databases, which yielded 76 literatures that met the inclusion and exclusion criteria. These literatures took the quantitative indicators of physical fitness as the outcome variables in 3–6-year-old children. This review found that of these 76 literatures analyzed, 71.1% came from Europe and 89.5% were published after 2010. The results showed six physical fitness test batteries, with the assessing FITness in PREschoolers (PREFIT) battery is the most widely used, and specific test items such as body mass index (BMI), standing long jump, handgrip, one-leg stance, sit and reach, 20 m shuttle run test (SRT)-PREFIT, and 4 × 10 m SRT are widely used in corresponding components. Therefore, we recommend that an international standard for some specific test items should be developed for preschool children to facilitate more widespread adoption and promote physical fitness assessment for preschool children.

## Introduction

Physical fitness refers to the ability of the various body systems to work together efficiently to perform daily activities and stay healthy (1). Physical fitness is considered an important indicator of child growth, development, and health status (2, 3), and has been indicated to be associated with academic achievement and cognitive functions (4, 5). Physical fitness is typically measured using five health-related (body composition, cardiovascular fitness, flexibility, muscular endurance, and strength) and six skill-related components (agility, balance, coordination, power, reaction time, and speed) (1). These components can provide information on the functioning and current health status of all body systems, and thus physical fitness assessment plays an important role in the daily health management and evaluation of children's growth and development. Especially because of the global decline in physical fitness and physical activity of children and adolescents (6, 7), the physical fitness assessment of children and adolescents has become increasingly significant.

In general, physical fitness assessment contains laboratory-based tests and field-based tests. Field-based tests have been used for many years to collect fitness data on thousands of individuals at a relatively lower cost and shorter time than laboratory-based tests, which are costly and time-consuming (8). Field-based physical fitness assessment methods for children (5+ years old) and adolescents are already well developed, with a number of established physical fitness test batteries, such as ALPHA-FIT (6), EUROFIT (7), and FITNESSGRAM (9), already being used internationally (10, 11). In contrast, despite the preschool age being a critical stage for basic motor skill development and physical adaption (12), and the research enthusiasm for physical fitness in preschool children, there are few internationally available physical fitness assessment batteries for preschoolers. In 2015, Assessing FITness in PREschoolers (PREFIT) was proposed for children at age of 3–5 years old (13), and its reliability, validity, objectivity, and feasibility were also reported (14). In addition, China released a national physical fitness test manual for preschool children in 2000 (15), however, there is no update to the test protocol since then. And even more, some countries still do not have an official physical fitness test battery applicable to preschool children. In Japan, for example, a unified assessment tool called New Physical Fitness Test has been developed for people aged 6–79 years old, but a similar tool for preschool children remains to be developed.

Given the growing concern about the physical fitness and growth/development of preschool children, it is necessary to know the current state of physical fitness assessment of preschool children worldwide to identify the research gaps that need to be addressed and to facilitate the development of physical fitness assessment for preschool children. However, there is no information available in the literature about what kind of field-based physical fitness test batteries and specific items are used for preschool children worldwide. Therefore, a scoping review was conducted in order to systematically map the research in this area, and the following questions were explored: (1) What kind of field-based physical fitness test batteries are used for preschool children worldwide; (2) Which specific assessment items are widely used in each physical fitness components; (3) What is the research gap on physical fitness assessment methods for preschool children; (4) What aspects should future studies aim to address.

## Materials and methods

Scoping reviews aim to provide an overview of existing literature usually without assessing the quality of included studies, to identify key concepts, knowledge gaps, and types of evidence in evolving research areas (16, 17). Thus, a scoping review was conducted to synthesize the current worldwide physical fitness assessment methods in preschool children to facilitate more widespread adoption and promote physical fitness assessment for preschool children. This scoping review followed the Preferred Reporting Items for Systematic Reviews and Meta-Analyses extension for scoping reviews (PRISMA-ScR) Statement (18) and was registered in the PROSPERO International Prospective Register of Systematic Reviews (registration number: CRD42021244173).

### Search strategy

PubMed, focusing on clinical and medical journals, is freely accessible and the optimal tool in biomedical electronic research. Web of Science, focusing on science, are databases that used a stricter proximity search to force the search to consider relevant words together, and covers the oldest publications, which can be traced back to 1900 (19). Combined with database features and this study topic, a literature search of these two electronic databases (PubMed and Web of Science) was conducted in April 2022. The search comprised all fields using the following search keywords/terms in PubMed: (“physical fitness” OR “health-related fitness” OR “motor-related fitness” OR “skill-related fitness”) AND (“preschool children” OR “preschooler” OR “early childhood” OR “children aged 3–6 years” OR “young children” OR “kindergartners”). Search string in Web of Science [ALL=(“physical fitness” OR “health-related fitness” OR “motor-related fitness” OR “skill-related fitness”)] AND ALL=[“preschool children” OR “preschooler” OR “early childhood” OR “children aged 3-6 years” OR “young children” OR “kindergartners”) and Articles (Document Types) and English (Languages). Additional studies were also identified from the reference lists of the review articles retrieved.

### Inclusion and exclusion criteria

Studies retrieved from the two databases were selected based on the same search terms. Title and abstract screening were performed and then an eligibility review of the full text was conducted according to the following criteria: (1) original literature in English published before 25 April 2022; (2) the population is preschool children (3–6 years old), without disease or disability; (3) physical fitness is measured by field-based methods and more than one quantitative indicator of physical fitness as the outcome variable, details are shown in Table 1.

**Table 1:** The inclusion and exclusion criteria.

**Content**	**Inclusion**	**Exclusion**
Population	preschool children (3-6 years old)	with disease or disability
Outcomes	Physical fitness is measured by field-based methods, and more than one quantitative indicator of physical fitness as the outcome variable	Reference value study of the physical fitness battery development team on a specific test item.
Publication range	published before April 25, 2022	-
Publication type	Article	-
Publication langue	English	Not published full text in English.

### Screening and selection process

**Figure 1:**
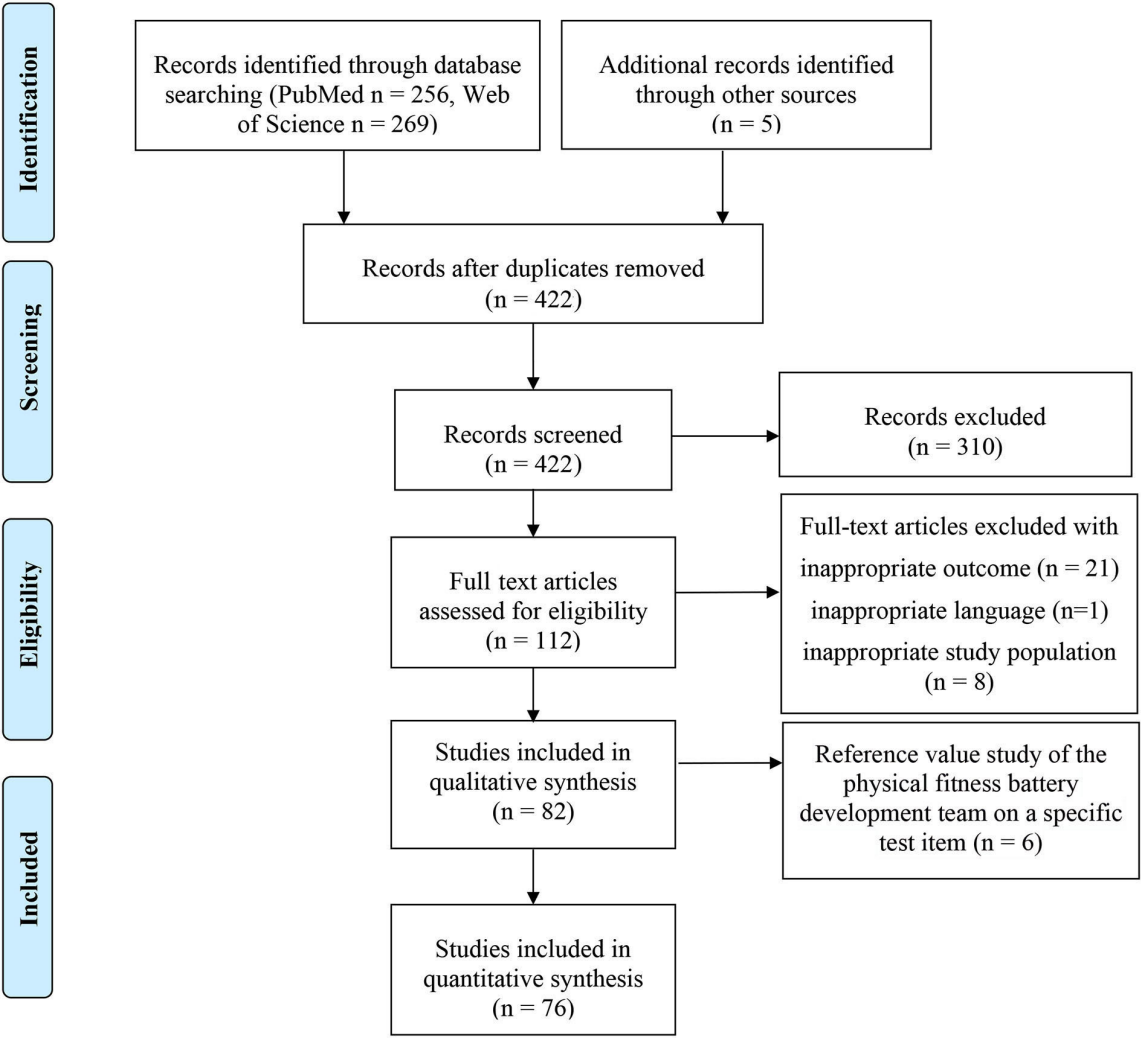
Flow diagram of the study selection.

A total of 468 records were found through electronic database searching (*n* = 525) and additional relevant studies were found from other sources (*n* = 5). The information of all kinds of literature was imported into the Excel file, and then the duplicates were removed, and a total of 422 articles were found. After reviewing titles and abstracts, 112 full-text articles were assessed for eligibility, in accordance with these two criteria: the outcome includes physical fitness field-based test battery or more than one test item and the population is preschool children. The above process was carried out by two co-authors independently, then discussed the dissent and updated the selection result. Then, 82 articles met the inclusion criteria, of which 6 articles are reference value studies from the physical fitness battery development team on a specific test item. Therefore, we did exclude them from the final analysis. Finally, 76 full-text articles were included in the quantitative synthesis. During the screening process, the reasons for inclusion and exclusion of each article are required to be recorded in Excel tables to facilitate the management of the screening process and result update. Figure 1 provides the screening process of the studies.

### Data charting

The two co-authors jointly developed a data chart in Microsoft Excel to determine that the following variables were extracted from the selected studies: title, authors, first author's affiliation, country, publication year, sample age, physical fitness test battery, physical fitness test items, and abstract. These two co-authors performed literature selection and data extraction independently, and discussed the results, when disagreements occurred, a third co-author was involved in making the final decision.

## Results

After literature screening and selection, 76 original studies that used field-based physical fitness tests were summarized. Of these, 47 studies referenced one field-based physical fitness battery. The publications' country, year, and the characteristic of the field-based physical fitness test battery and specific items are presented below.

### Summary of the included study

**Figure 2:**
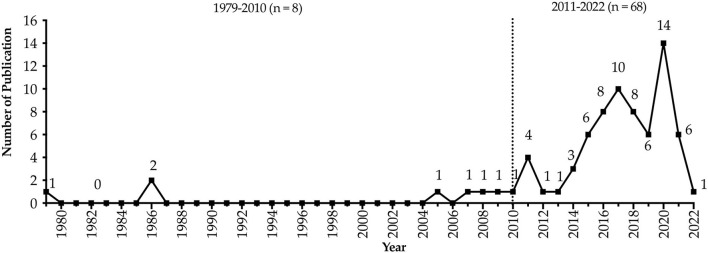
Yearly distribution of the number of publications.

Bibliometric statistics were carried out according to the country of origin (Table 2) and the year of publication (Figure 2), respectively. As shown in Table 2, of the 76 literatures examined, 54 (71.1%) were from Europe (14, 20–72), 16 (21.0%) from Asia (73–88), and 6 (7.9%) from the Americas (89–94). As shown in Figure 2, most studies on the physical fitness of preschool children were published after 2011 and accounted for 68 (89.5%) of the total inclusions, especially after 2015, there are more than six publications per year.

**Table 2:** The distribution of publications by country of origin.

**Continent**	**Country**	**Number**	**Continent**	**Country**	**Number**
Europe	Spain	21	Asia (*n* = 16)	China	11
(*n* = 54)	Sweden	6		Japan	2
	Switzerland	5		Israel	1
	Germany	4		Iran	1
	Norway	2	Americas (*n* = 6)	Turkey	1
				Canada	3
	Austria	2		Chile	2
	Italy	3		Colombian	1
	Poland	2			
	Serbia	2			
	Czech Republic	2			
	Croatia	1			
	Estonia	1			
	Portugal	1			
	Russia	1			
	United Kingdom	1			

### Field-based test battery

A total of six physical fitness test batteries from different countries were used in 47 studies on preschool children. The test items, corresponding components, and adoption frequency of six physical fitness test batteries are summarized in Table 3.

**Table 3:** Component-based contents of the physical fitness batteries for preschool children.

**Test battery (citation number)**	**Region (start year)**	**Age**	**Health-related**				**Skill-related**					**Reliability report**	**Country (citations)**
			**Body composition**	**Cardio** **vascular fitness**	**Muscular strength**	**Flexibility**	**Agility-speed**	**Power**	**Balance**	**Coordination**	**Reaction time**		
Assessing FITness in PREschoolers PREFIT (*n* = 24)	Spain (2015)	3–5 years	①Height, weight, BMI	③20 m SRT	④Handgrip strength	-	⑤4 × 10 m SRT	⑥Standing long jump	⑦One-leg stance test	-	-	Good reliability, except for one-leg stance	Spain (*n* = 11) (14, 20–28, 72)
													Sweden (*n* = 6) (29–32, 65, 66)
			②Waist circumference										China (n = 1) (73)
													Estonia (*n* = 1) (36)
													Norway (*n* = 1) (33)
													Italy (*n* = 1) (37)
													Chile (*n* = 1) (89)
													Turkey (*n* = 1) (88)
													Serbia (n = 1) (38)
Chinese National Physical Fitness Measurement (*n* = 11)	China (2000)	3–6 years	①Height, weight, BMI	-	-	②Sit and reach	③2×10 m SRT	④Standing long jump	⑥Balance beam walk	⑦5 m Jumping on 2 feet	-	Good reliability, except for balance beam walking	China (*n* = 11) (73–80, 84–86)
								⑤Tennis ball throw					
The fitness test battery by Latorre (*n* = 6)	Spain (2015)	3–6 years	-	①10 × 20 m	-	-	②20 m run	③Standing long jump	④Balance stand Test	-	⑤Ruler drop test	Good reliability	Spain (n = 6) (39–44)
Karlsruher Motorik-Screening für Kindergartenkinder (KMS 3-6) (*n* = 4)	Germany (2004)	3–6 years	-	-	-	①Stand and reach	-	②Standing long jump	③One-legged stance 1mins	④15 s Side-to-side jumps	-	Good reliability (in German)	Germany (*n* = 2) (45, 46)
													Austria (*n* = 2) (34, 35)
the “Fuprecol kids” battery (n = 1)	Colombian (2019)	3–5 years	①Height, weight, BMI	⑤20 m SRT-PREFIT	④Handgrip strength	⑤Sit and reach	⑥4 × 10 m SRT	⑦Standing long jump	-	-	-	Good reliability	Colombian (n = 1) (90)
			Waist circumference										
Kinderturn-Test (*n* = 1)	Germany (2006)	3–6 years	-	-	-	①Stand and reach	-	②Standing long jump	③Balance beam walk	④Side-to-side jumps	-	Good reliability, except for balance beam walking (in German)	Germany (*n* = 1) (56)

BMI, Body Mass Index; SRT, Shuttle Run Test.

Regarding the inclusion frequency of physical fitness components, in these six batteries, the order of frequency in skill-related components is (power (6/6ths), balance (5/6ths), agility-speed (4/6ths), coordination (3/6ths), and reaction time (1/6ths). The frequency of health-related components is flexibility (4/6ths), body composition (3/6ths), cardiovascular fitness (3/6ths), muscular strength (2/6ths), and muscular endurance (0/6ths).

The six test batteries and the citations by country are listed as below:

1. Assessing FITness in PREschoolers (PREFIT) (14) includes seven test items covering three health-related (body composition, cardiovascular fitness, and muscular strength) and three skill-related components (agility-speed, power, and balance). PREFIT is widely used in eight countries: Spain (n = 11) (14, 20–28, 72), Sweden (n = 6) (29–32, 65, 66), China (n = 1) (73), Estonia (n = 1) (36), Norway (n = 1) (33), Italy (n = 1) (37), Chile (n = 1) (89), Turkey (n=1) (88), and Serbia (n = 1) (38).
2. Chinese National Physical Fitness Measurement (CNPFM-Pre) (15) consists of seven test items covering two health-related (body composition and flexibility) and four skill-related components (agility-speed, power, balance, and coordination). The relevant studies were all conducted in China (n = 11) (73–80, 84–86).
3. The fitness test battery by Latorre Román et al. (44) includes five test items covering one health-related (cardiovascular fitness) and four skill-related components (agility-speed, power, balance, and reaction time). This test battery is used in Spain (*n* = 6) (39–44).
4. Karlsruher Motorik-Screening für Kindergartenkinder (KMS 3-6) (95) consists of four test items: stand and reach test for flexibility, standing long jump test for power, one-legged stance test for balance, and 15 s side-to-side jumps test for coordination. KMS 3–6 is adopted in Germany (*n* = 2) (45, 46) and Austria (*n* = 2) (34, 35).
5. The “Fuprecol kids” battery (90) consists of seven test items covering four health-related (body composition, cardiovascular fitness, muscular strength, and flexibility) and two skill-related components (agility-speed and power). This test battery is used in Colombia (*n* = 1) (90).
6. Kinderturn-Test (96) consists of four test items covering one health-related (flexibility) and three skill-related components (power, balance, and coordination) and is used in Germany (*n* = 1) (56).

### Component-based specific test item

A total of 20 health-related and 25 skill-related specific test items were found in the 65 studies. Table 4 presents all test items and citation numbers according to the component categories. Body mass index (BMI), 20 m shuttle run test (SRT-PREFIT), handgrip strength, sit and reach, and sit-ups are mostly used to reflect each health-related component. The standing long jump, 4 × 10 m SRT, one-leg stance, side-to-side jumps, and ruler drop test are the most popular test method in each skill-related physical fitness component.

**Table 4:** Summary of physical fitness specific test items for preschool children.

**Domin (*n*)**	**Sub-domin (*n*)**	**Specific tests**	**Use frequency**	**Sub-domin (*n*)**	**Specific tests**	**Use frequency**
Health-related (*n* = 20)	Body composition (*n* = 4)	**Height, weight, BMI**	**70**	Muscular strength (*n* = 1)	**Handgrip strength**	**32**
		Waist circumference	17			
		%body fat	16	Flexibility (*n* = 3)	**Sit and reach**	**21**
		Skinfold thickness	8		Stand and reach	2
	Cardiovascular fitness (*n* = 8)	**20 m SRT-PREFIT**	**21**		Sitting trunk exion	1
		20 m SRT-Original	11	Muscular endurance (*n* = 4)	**Sit-ups**	**5**
		10 × 20 m	6		Bent-arm hang	2
		Modified step test	3		Climbing wall bars	1
		Mini-Cooper test (6 min)	2		Pull up	1
		3 min SRT	2			
		6-min walk test	1			
		600 m-run	1			
Skill-related (*n* = 25)	Power (*n* = 5)	**Standing long/broad jump**	**56**	Balance (*n* = 3)	**One-leg stance**	**26**
		Tennis ball throw	9		Balance beam walk	13
		Overhead medicine ball (1 kg) throw	3		Balance plateform	2
		Pushing a medicine ball (1 kg) with 2 hands	2	Coordination (*n* = 5)	**Side-to-side jumps**	**7**
		Sargent jump test	2		5 m Jumping on 2 feet	7
	Agility-speed (*n* = 11)	**4** **× 10 m SRT**	**20**		7 m Jumping on 2 feet	2
		20 m run	11		Hopping on one leg	2
		2 × 10 m SRT	11		7 m Jumping on 1 foot	1
		Obstacle course	7	Reaction time (*n* = 1)	**Ruler drop test**	**5**
		25 m run	6			
		30 m run	2			
		10 × 5 m SRT	1			
		50 feet (15.2 m) SRT	1			
		a jump over and crawl	1			
		4 × 9 m SRT	1			
		2 × 9 m SRT	1			

BMI, Body Mass Index; SRT, Shuttle Run Test.

## Discussion

The aim of this study was to review physical fitness studies conducted for preschool children, as well as to summarize the field-based physical fitness batteries and specific items that have been adopted for preschool children worldwide. Overall, 76 literatures were ultimately included in the final summary analysis after screening and eligibility, and of these 76 literatures, a total of six physical fitness test batteries (Table 3), 20 health-related and 25 skill-related specific test items (Table 4) were adopted.

There is no doubt that physical fitness has always been an important part of life. Since the 1960's, field-based physical fitness test batteries for children (5+ years old) and adolescents have been utilized, and numerous review articles have reported on these widely used batteries (11, 97, 98). However, within the scope of our search, there are only eight publications related to preschool children's physical fitness assessment prior to 2010. Since 2014, enthusiasm for physical fitness assessment of preschool children has been increasing, and the number of relevant publications reached 14 by 2020. The change in the yearly distribution of publications' number might be due to the increasing importance of early childhood education in many western countries over the past two decades, which means the increase in early childhood institutions and the professionalization of kindergarten teachers and preschool teachers. This trend may lead to a boost in publications on this topic. From the distribution of publications in each country (Table 2) and the region of six physical fitness test batteries (Table 3), it can be observed that Spain and China have the most publications, probably because these two countries released two [PREFIT (14) and the Physical Fitness Battery by Cadenas-Sanchez et al. (14)] and one [CNPFM-Pre (15)] physical fitness assessment batteries, respectively, and the reliabilities of assessment batteries were reported in English.

As presented in Table 3, Of these 76 literatures reviewed, there are six field-based physical fitness test batteries specifically designed for preschool children. Overall, skill-related physical fitness components are more frequent than health-related components contained in these batteries. This might be explained that the preschool age is a critical period for the acquisition of fundamental motor skills (12), and at this stage, it is important to monitor motor skill development. Regarding the reliability, the reliabilities of all these six batteries were presented, four [PREFIT (14), CNPFM-Pre (75), the Physical Fitness Battery by Latorre Román et al. (44), and the Fuprecol kids battery (90)] in English, and two [KMS 3–6 (95) and Kinderturn-Test (96)] in German. The PREFIT study group indicated that all tests of the PREFIT battery are feasible and highly reliable, except for the one-leg stance test which requires further study (14). Fang and Ho (75) demonstrated that all test items in CNPFM-Pre had good relative reliability, and only the balance beam walking test showed low reliability, which is consistent with the kinderturn test that has good reliability for all items except for the balance beam walk (96). Latorre Román et al. (44) presented that the Fitness Test Battery showed adequate test-retest reliability. “Fuprecol kids” assessment battery (90) and KMS 3–6 (95) were both noted to be reliable and feasible for preschoolers. In addition, it is notable that the “PREFIT” battery is the only one that includes an English assessment manual (99) and criteria for Spanish children (23), which makes it easier for other researchers to follow and may explain why the PREFIT battery is the only one adopted by researchers from different countries.

Physical fitness contains 11 components (1), and in the field-based test battery design and practical applications, researchers may select appropriate items based on the order of importance or study relevance rather than including them all. As reported in Table 4, muscular endurance, coordination, and reaction time are less emphasized compared to other components. This may be because these abilities are not well developed in the early childhood stage, although this could also be related to the difficulty of conducting the tests, and the relative complexity of the methods for young children to understand. It was also worth noting that a total of 45 different test methods were used in the 76 studies reviewed here, and these included several different methods for each physical fitness component. Agility speed, for example, was measured using 11 different specific tests. However, having a diverse selection of methods might not facilitate cross-sectional comparisons among scholars or longitudinal observation of trends for a particular group. Therefore, it might make sense to select one or two items for each component that have high reliability and validity and are widely accepted and focus future studies on these.

The standardization of physical fitness assessment and evaluation criteria is essential for the regular monitoring of preschool children's physical fitness, as well as for international collaboration in the study of preschool children's physical fitness. Of the 76 papers reviewed here, no study suggests a global standard of one physical fitness battery or a specific item. Nor are there any international collaborative studies that use the same test battery or some unified test items. Based on the results of this review, there are very few batteries in widespread use around the world, and developing a standardized physical fitness assessment and evaluation criteria is not yet feasible. However, there are test items in each component that are more commonly used, such as the standing long jump, handgrip, one leg stance, and 20 m SRT-PREFIT. Therefore, we recommend that future studies refer to Tomkinson's approach (100) and develop an international standard for a specific test item for preschool children based on existing research data. In addition, it is suggested that researchers co-work on establishing one applicable physical fitness battery for preschool children in their country or continent. Also, further research could also initiate a traditional systematic review covering multiple languages and focusing on validated physical fitness batteries. These research efforts may promote the standardization of physical fitness assessment for preschool children, which in turn could be beneficial in promoting the physical activity levels and fitness performance of preschool children.

There are some limitations to this study. First, this scoping review only covers English articles in the two main databases and may thus have missed studies or policies that have been published in a different language. Our results may therefore be disproportionately influenced by English-speaking countries and may not accurately represent the global state of physical fitness assessment for preschool children. Second, this study excluded the fundamental motor skill measurement tools that are also widely used to evaluate motor skill development. This was done as a physical fitness assessment (including health- and skill-related components) is somewhat different from a fundamental motor skill assessment. A physical fitness assessment is a simpler and more quantitative indicator and may therefore play a greater role in daily monitoring and study. Nevertheless, this is the first study to provide baseline reference information for researchers aiming to study the growth and development of young children. In addition, we identify current research gaps and point to future research directions that are required to address these gaps to develop physical fitness assessment protocols for preschool children.

## Conclusions

This review found that most literatures were published after 2010 and are concentrated in Europe. only the PREFIT battery has been adopted for preschool children in seven other countries, and specific test items such as BMI, standing long jump, handgrip, one-leg stance, sit and reach, 20 m SRT-PREFIT, and 4 × 10 m SRT are widely used in corresponding components. Therefore, we recommend that an international standard for some specific test items should be developed for preschool children to facilitate more widespread adoption and promote physical fitness assessment for preschool children.

## Author contributions

KS, RM, and DK: conceptualization. DK and RM: methodology and formal analysis. DK: writing–original draft preparation. SS, HK, and YK: writing–review and editing. KS: supervision, project administration, and funding acquisition. All authors have read and agreed to the published version of the manuscript.

## Funding

This work was supported by research grants from the Institute of Health and Sports Science and Medicine, Juntendo University; Joint Research Program of Juntendo University, Faculty of Health and Sports Science; JSPS KAKENHI Grant Number JP20K11450.

## Conflict of interest

The authors declare that the research was conducted in the absence of any commercial or financial relationships that could be construed as a potential conflict of interest.

## Publisher's note

All claims expressed in this article are solely those of the authors and do not necessarily represent those of their affiliated organizations, or those of the publisher, the editors and the reviewers. Any product that may be evaluated in this article, or claim that may be made by its manufacturer, is not guaranteed or endorsed by the publisher.
